# The crystal structure of the RhoA–AKAP-Lbc DH–PH domain complex

**DOI:** 10.1042/BJ20140606

**Published:** 2014-11-14

**Authors:** Kamal R. Abdul Azeez, Stefan Knapp, João M. P. Fernandes, Enno Klussmann, Jonathan M. Elkins

**Affiliations:** *Structural Genomics Consortium, Oxford University, Old Road Campus Research Building, Old Road Campus, Roosevelt Drive, Oxford OX3 7DQ, U.K.; †Target Discovery Institute, Oxford University, NDM Research Building, Old Road Campus, Roosevelt Drive, Oxford OX3 7FZ, U.K.; ‡Max Delbrück Center for Molecular Medicine (MDC), Robert-Rössle-Str. 10, 13125 Berlin, Germany

**Keywords:** AKAP13, AKAP-Lbc, GTPase, RhoGEF, AKAP, A-kinase-anchoring protein, DH, Dbl-homologous, GEF, guanine-nucleotide-exchange factor, ITC, isothermal titration calorimetry, LARG, leukaemia-associated RhoGEF, MANT, *N*-methylanthraniloyl, MAPK, mitogen-activated protein kinase, PH, pleckstrin homology, PKA, protein kinase A, PRKD1, protein kinase D1, TEV, tobacco etch virus

## Abstract

The RhoGEF (Rho GTPase guanine-nucleotide-exchange factor) domain of AKAP-Lbc (A-kinase-anchoring protein-Lbc, also known as AKAP13) catalyses nucleotide exchange on RhoA and is involved in the development of cardiac hypertrophy. The RhoGEF activity of AKAP-Lbc has also been implicated in cancer. We have determined the X-ray crystal structure of the complex between RhoA–GDP and the AKAP-Lbc RhoGEF [DH (Dbl-homologous)–PH (pleckstrin homology)] domain to 2.1 Å (1 Å=0.1 nm) resolution. The structure reveals important differences compared with related RhoGEF proteins such as leukaemia-associated RhoGEF. Nucleotide-exchange assays comparing the activity of the DH–PH domain to the DH domain alone showed no role for the PH domain in nucleotide exchange, which is explained by the RhoA–AKAP-Lbc structure. Comparison with a structure of the isolated AKAP-Lbc DH domain revealed a change in conformation of the N-terminal ‘GEF switch’ region upon binding to RhoA. Isothermal titration calorimetry showed that AKAP-Lbc has only micromolar affinity for RhoA, which combined with the presence of potential binding pockets for small molecules on AKAP-Lbc, raises the possibility of targeting AKAP-Lbc with GEF inhibitors.

## Abstract 2

The crystal structure of the RhoGEF domain of AKAP-Lbc in complex with RhoA combined with nucleotide exchange assays explain differences to related RhoGEF proteins and allow the possibility of targeting the AKAP-Lbc RhoGEF domain with small molecules.

## INTRODUCTION

AKAP-Lbc (also known as AKAP13) is a member of the A-kinase-anchoring proteins [1,2]. AKAPs are diverse in sequence and domain composition, but have a common feature of binding the PKA (protein kinase A) regulatory subunit and controlling the localization of PKA [3–5]. The protein scaffolding mediated by AKAP-Lbc includes KSR1 (kinase suppressor of Ras 1), PKA, RAF, MEK [MAPK (mitogen-activated protein kinase)/ERK (extracellular-signal-regulated kinase) kinase], IKKβ [IκB (inhibitor of nuclear factor κB) kinase β] [6], protein kinases D, C [7,8] and N [9], SHP2 (Src homology 2 domain-containing protein tyrosine phosphatase 2) [10], Hsp20 (heat-shock protein 20) [11], p38 MAPK and 14-3-3 proteins [12–14]. AKAP-Lbc exists as at least three splice variants, however, all contain a conserved RhoGEF (Rho GTPase guanine-nucleotide-exchange factor) domain. RhoGEF domains decrease the affinity of a Rho GTPase for GDP so that GTP, present in higher concentrations in the cell, can bind. Thus RhoGEFs activate Rho GTPases allowing extracellular stimuli to promote Rho–GTP formation which initiates signalling pathways inside the cell.

RhoGEF domains are composed of a DH (Dbl-homologous) domain followed by a PH (pleckstrin homology) domain. This tandem DH–PH domain is a conserved unit in most RhoGEF proteins [15]. The DH domain is essential, but the role of the PH domain is variable. In some RhoGEFs, the PH domain controls protein localization through binding to phospholipids [16], or possibly in some cases to other molecules. However, for some RhoGEFs, the PH domain also contributes to the activity of the DH domain in catalysing nucleotide exchange, such as in the examples of PDZ-RhoGEF (ARHGEF11) [17,18], Dbs (MCF2L) [19–21], p115-RhoGEF [22] or leukaemia-associated RhoGEF (LARG and ARHGEF12) [23]. In the case of PDZ-RhoGEF, the PH domain was more important for nucleotide exchange with CDC42 than with RhoA [17]. In some cases, activated RhoA interacts with the PH domains as a form of positive feedback through a mechanism not involving alteration of the nucleotide-exchange rate [24].

**Figure 1:**
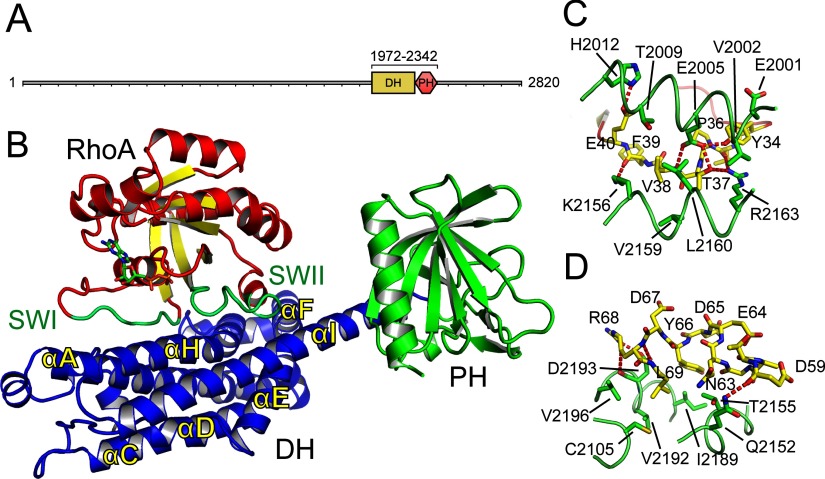
Structure of AKAP-Lbc (**A**) Protein domains of AKAP-Lbc (AKAP13). The range of the construct used for structure determination is shown above the DH and PH domains. (**B**) Overview of the complex between RhoA–GDP and the DH–PH domains of AKAP-Lbc. RhoA is shown with red α-helices and yellow β-strands, and with the switch I and switch II loops in green. AKAP-Lbc is shown with its DH domain in blue and PH domain in green. (**C**) The binding of the RhoA switch I loop. The switch I loop is shown in yellow, with the AKAP-Lbc DH domain shown in green. (**D**) The binding of the RhoA switch II loop, coloured as for (**C**).

AKAP-Lbc has a RhoGEF DH–PH domain between residues 1972 and 2342 (Figure 1A). Its PH domain was shown to be dispensable for activation of Rho, but important for the localization and transformative activity of AKAP-Lbc [25]. The region C-terminal to the DH–PH domain is involved in scaffolding PRKCH [also known as PKCη (protein kinase Cη)] and PRKD1 (protein kinase D1) to activate PRKD1 [8]. This C-terminal region is altered in oncogenic AKAP-Lbc [26].

AKAP-Lbc has been shown to have a role in the development of cardiac hypertrophy [3,7,27,28]. In addition, a truncated form of AKAP-Lbc has been identified as an oncoprotein [29,30]. Evidence suggests that this oncogenic activity of AKAP-Lbc is through activation of the Rho pathway, via the RhoGEF activity of AKAP-Lbc [31,32]. An association between AKAP-Lbc genetic variants and high-risk familial breast cancer has also been detected [33]. These disease links involving the RhoGEF activity of AKAP-Lbc suggest that inhibitors of the RhoA–AKAP-Lbc interaction may be a worthwhile line of investigation for treatment of cardiac hypertrophy, pulmonary arterial hypertension [34] and possibly for cancers. For example, AKAP-Lbc is absent in normal liver, but expressed in hepatocarcinoma, where transformation was blocked by a Rho inhibitor [35]. The possibility in principle of targeting a GTPase–GEF interface has been demonstrated for Rac1–Tiam1 [36,37] and for RhoA–LARG [38]. To support these investigations on RhoA–AKAP-Lbc and to investigate the mechanism of activation of RhoA by AKAP-Lbc we have determined the X-ray crystal structure of RhoA–GDP in complex with the DH–PH tandem domain of AKAP-Lbc, compared it with the structure of the DH domain alone, and analysed the binding affinities and rates of nucleotide exchange in the RhoA–AKAP-Lbc complex.

## MATERIALS AND METHODS

### Cloning

DNA for RhoA (gi|10835049) or AKAP13 (gi|31563330, NP_006729.4, AKAP13 isoform 1) was PCR amplified and subcloned into an in-house pET-based vector pNIC28-Bsa4 [39] using ligation-independent cloning. The DNA template for RhoA was obtained from the Mammalian Gene Collection (IMAGE Consortium Clone ID 4102976). The resulting constructs expressed the desired proteins with N-terminal His_6_-tags and TEV (tobacco etch virus) protease tag cleavage sites (extension MHHHHHHSSGVDLGTENLYFQ*SM). AKAP13 DH domain point mutant constructs were created by PCR using the His_6_-tag DH–PH domain expression construct as a template. All mutations were verified by DNA sequencing.

### Protein expression and purification

RhoA and AKAP13 DH–PH constructs were transformed into *Escherichia coli* BL21(DE3) competent cells containing the pRARE2 plasmid from the commercial Rosetta strain, and the transformants used to inoculate 50 ml of LB medium containing 50 μg/ml kanamycin and 34 μg/ml chloramphenicol. These cultures were incubated overnight at 37°C. These cultures were used to inoculate larger cultures of TB medium (RhoA) or LB medium (AKAP13) with 40 μg/ml kanamycin and grown at 37°C until an *D*_600_ of 1.0 (RhoA) or 0.4–0.5 (AKAP13) was reached. The temperature was then reduced to 20°C and the cultures induced with 0.5 mM IPTG. Expression was continued overnight. The cells were harvested by centrifugation, resuspended in binding buffer (500 mM NaCl, 50 mM Hepes, pH 7.5, 5% glycerol, 20 mM imidazole, pH 7.5, and 0.5 mM TCEP) and frozen until further use.

For purification, the cells were thawed and lysed by sonication on ice. PEI (polyethyleneimine) was added to a final concentration of 0.15% and the cell debris and precipitated DNA were spun down. RhoA and AKAP13 were each purified by passing the supernatants through a column of 5 ml of Ni^2+^-Sepharose resin (GE Healthcare). After washing, the protein was eluted with 25 ml of binding buffer containing 250 mM imidazole. The His_6_-tags were removed using TEV protease. AKAP13 was then mixed in equimolar ratio with RhoA, and the complex was concentrated and injected on to a S75 16/60 gel filtration column pre-equilibrated into GF buffer (50 mM Hepes, pH 7.5, 500 mM NaCl, 5% glycerol and 0.5 mM TCEP). After passing through a gravity column of 5 ml of Ni^2+^-Sepharose to remove impurities, the RhoA–AKAP13 complex was concentrated to 5.1 mg/ml. Protein concentrations were measured by UV absorbance, using the calculated molecular masses and estimated molar absorption coefficients, using a NanoDrop spectrophotometer (Thermo Scientific). The constructs of the AKAP13 DH domain and the DH domain point mutations were used to express and purify protein following a similar protocol.

### Crystallization and data collection

RhoA–AKAP13 domain crystals grew by vapour diffusion at 4°C from a mixture of 50 nl of protein and 100 nl of a well solution containing 0.2 M ammonium sulfate, 0.1 M Bis-Tris, pH 5.5, and 25% (w/v) PEG 3350. Crystals were equilibrated into reservoir solution plus 25% ethylene glycol before freezing in liquid nitrogen. Data were collected at 100 K at the Diamond Synchrotron beamline I02. Data collection statistics can be found in Table 1.

**Table 1:** Data collection and refinement statistics Values within parentheses refer to the highest resolution shell.

Parameter	RhoA–AKAP-Lbc DH–PH	AKAP-Lbc DH
PDB code	4D0N	4D0O
Space group	*P*2_1_2_1_2_1_	*P*2_1_2_1_2_1_
Number of molecules in the asymmetric unit	1	2
Unit cell dimensions *a*, *b*, *c* (Å)	82.3, 86.6, 116.8	52.1, 94.8, 109.0
Data collection		
Resolution range (Å)	82.32–2.10 (2.16–2.10)	42.13–2.75 (2.90–2.75)
Unique observations	49042 (3824)	14660 (2101)
Average multiplicity	3.4 (2.9)	6.5 (6.5)
Completeness (%)	99.3 (95.8)	100.0 (100.0)
*R*_merge_	0.06 (0.45)	0.21 (1.18)
Mean (*I*)/σ(*I*)	8.2 (1.6)	7.2 (2.1)
Mean CC(1/2)	0.998 (0.784)	0.992 (0.530)
Refinement		
*R*-value, *R*_free_ (%)	21.1, 23.9	24.0, 30.8
RMSD from ideal bond length (Å)	0.006	0.005
RMSD from ideal bond angle (°)	1.07	0.887

DH domain crystals grew under identical conditions [50 nl of protein and 100 nl of a well solution containing 0.2 M ammonium sulfate, 0.1 M Bis-Tris, pH 5.5, and 25% (w/v) PEG 3350] and were equilibrated into reservoir solution plus 20% ethylene glycol before freezing in liquid nitrogen. Data were collected at 100 K at the Diamond Synchrotron beamline I03.

### Structure determination

The diffraction data were indexed and integrated using MOSFLM [40] and scaled using AIMLESS [41]. The structure was solved by molecular replacement using PHASER [42] and the structures of RhoA (PDB code 1XCG [17]) and p115-RhoGEF (PDB code 3ODW [43]) as search models. There was one molecule of the RhoA–AKAP-Lbc complex in the asymmetric unit. The model was built using Coot [44] and refined with REFMAC5 [45]. Rebuilding and refinement resulted in the final model. The model was validated using MOLPROBITY [46]. All structure figures were created using PyMOL (Schrödinger).

### ITC (isothermal titration calorimetry)

Measurements were made on a MicroCal iTC200 (GE Healthcare) at 15°C. For measurements in the presence of GDP, all proteins were dialysed overnight into a buffer consisting of GF buffer with the addition of 50 μM GDP. For measurements in the absence of GDP, RhoA was incubated overnight with 5 mM ETDA and 6 units/ml alkaline phosphatase followed by gel filtration in GF buffer, and finally concentrated as before and dialysed overnight in GF buffer. For each measurement, the syringe was loaded with 0.6 mM RhoA (in the absence or presence of GDP) and 20×2 μl injections were made into the cell, which was filled with 0.04 mM of either AKAP-Lbc DH domain or AKAP-Lbc DH–PH domain. Data were analysed using MicroCal and Origin software. The results after fitting to a single-site model are shown in Table 2.

**Table 2:** ITC measurements Errors represent the error in the fit to the binding curve, not the overall error in the measurement.

	RhoA–GDP + DH	RhoA–GDP + DH–PH	RhoA + DH	RhoA + DH–PH
*N*	0.55	0.55	0.68	0.35
Δ*H* (kcal/mol)	20.2±2.0	7.6±0.9	12.7±0.5	49.6±10.2
TΔ*S* (kcal/mol)	26.4	13.9	66.9	55.6
*K*_d_ (μM)	21.3±2.0	18.1±2.3	10.2±0.7	33.9±3.0

### GDP/GTP exchange assays

RhoA was incubated with alkaline phosphatase to remove bound nucleotide and then purified by gel filtration under the same conditions as above. The nucleotide-free RhoA was incubated with MANT (*N*-methylanthraniloyl)-GDP (Life Technologies) for 15 min. The protein was then passed through a PD-10 column pre-equilibrated in GF buffer to remove excess MANT-GDP. Guanine-nucleotide-exchange assays were conducted in 20 μl aliquots in black 384-well low-volume plates (Greiner). The progress of the nucleotide exchange was monitored by fluorescence on a PheraStar plate reader (BMG Labtech) with excitation at 360 nm and detection at 440 nm. Exchange reactions were measured in GF buffer containing 10 mM MgCl_2_, with 0.5 μM RhoA–MANT-GDP and 50 μM unlabelled GMP-PNP, and in the presence or absence of varying concentrations of DH or DH–PH domain protein.

### Druggability score calculations

Protein structures for binding-site analysis were prepared using the protein preparation function in Maestro (Schrödinger). All protein chains except the chain of interest were deleted. Hydrogen atoms were added and missing side chains were rebuilt using Prime (Schrödinger). Druggability scores were calculated using SiteMap (Schrödinger), using the default settings as implemented in Maestro, using the OPLS_2005 force field definition. For calculation of SiteMap scores from bromodomains, the conserved water molecules that form part of the acetyl-lysine binding pocket were retained in the structure used in the analysis.

## RESULTS

### Structure determination

Bacterial overexpression constructs were prepared for RhoA (residues 1–184) and the DH–PH domain of AKAP-Lbc (residues 1972–2342, residue numbers refer to AKAP13 isoform 1, Genbank ID NP_006729.4) (Figure 1A and Table 3). After initial purification the proteins were mixed and passed through a size-exclusion chromatography column together. RhoA and the DH–PH domain eluted at the same time from the size-exclusion column. Both the DH–PH domain alone and the complex of RhoA and DH–PH domain appeared to be monomeric during size-exclusion chromatography.

**Table 3:** Protein expression constructs

	RhoA	AKAP-Lbc DH–PH domain	AKAP-Lbc DH domain
Residue range*	Met^1^–Gly^184^	Lys^1973^–Asp^2342^	Glu^1976^–Lys^2211^
Expression vector	pNIC28-Bsa4	pNIC28-Bsa4	pNIC28-Bsa4
Molecular mass (kDa)	20.9	43.4	27.9

*Sequences of the protein constructs that were used can be found in the Supplementary Online Material.

Crystals of the RhoA–DH–PH domain complex were obtained in the presence of GDP which allowed the structure to be determined by X-ray diffraction to 2.1 Å (1 Å=0.1 nm) resolution (Table 1). There was one molecule of a 1:1 RhoA(GDP)–DH–PH complex in the asymmetric unit (Figure 1B). The model was refined to a free *R*-factor of 23.8%. Residues 3–181 of RhoA and residues 1972–2340 of AKAP-Lbc (including the DH and PH domains) were resolved in the electron density.

### AKAP-Lbc binds RhoA via its switch I and switch II loops

**Figure 2:**
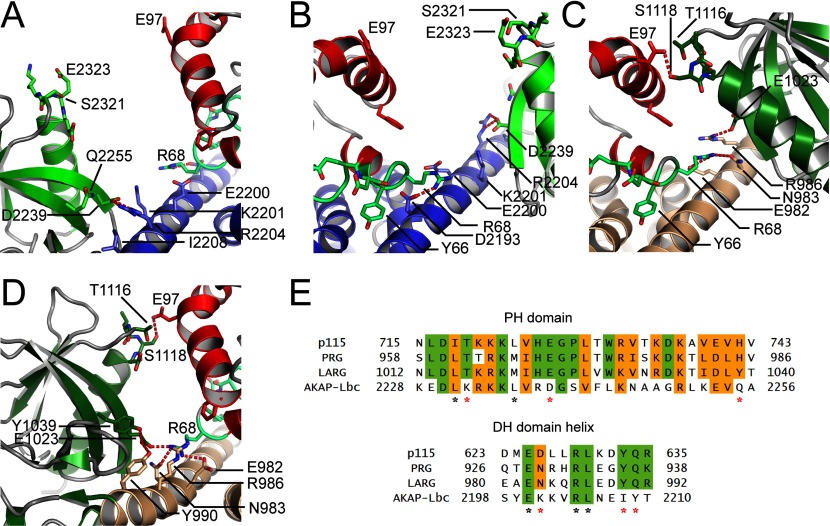
No contact between the AKAP-Lbc PH domain and RhoA (**A** and **B**) Two views of the separation between RhoA and the PH domain. Residues which in homologous proteins are involved in RhoA–PH domain interactions are shown as a stick representation. RhoA is coloured red, and the AKAP-Lbc DH and PH domains are coloured blue and green. (**C** and **D**) Two views, from equivalent orientations as (**A**) and (**B**), of the interaction between the PH domain of LARG and RhoA (PDB code 1X86). The LARG DH and PH domains are coloured brown and dark green. (**E**) Sequence alignment of the regions of the PH domain and DH domain potentially involved in RhoA–PH domain interactions, for AKAP-Lbc and three homologous RhoGEF domains, all three of which show an effect of the PH domain on nucleotide-exchange catalysis. Residues involved in the interface in LARG are indicated with asterisks below the alignment. For residue differences likely to be important for the lack of effect of the AKAP-Lbc PH domain, the asterisks are coloured red.

Overall, the structure resembles the general domain arrangement seen in previously published complexes between RhoA and other RhoGEFs. The AKAP-Lbc DH domain interacts with the switch I and switch II loops of RhoA, with a buried surface area at the interface of 1319 Å^2^ (Figure 2A). However, the closest relation to AKAP-Lbc that has been crystallized previously is p115-RhoGEF [43] with 31% sequence identity to AKAP-Lbc over the DH–PH domain (Supplementary Figure S1) and many of the residues involved in the interface with RhoA are not conserved in other RhoGEFs.

The RhoA switch I loop is bound between helices αA and αH of the DH domain (Figures 1B and 1C). At the C-terminal end of the loop, Glu^40^ forms a hydrogen bond with His^2012^ of the DH domain while the backbone carbonyl of Phe^39^ forms a hydrogen bond with the side chain of Lys^2156^. A central hydrophobic patch consisting of Thr^2009^, Val^2159^ and Leu^2160^ interacts with the RhoA residue Val^38^. At the N-terminal end of switch I, there is an extensive hydrogen-bonding network with the DH domain where the DH domain Glu^2005^ binds simultaneously the backbone nitrogen of RhoA Val^38^ and the side chains of Thr^37^ and Tyr^34^, while the DH domain Arg^2163^ also forms a hydrogen bond with Thr^37^ as well as the backbone carbonyl of Val^35^. An additional hydrophobic packing involves the aromatic ring of RhoA Tyr^34^ and the side chain of Glu^2001^.

The Rho switch II loop is bound in a similar pattern to switch I, with polar interactions between each end of the loop and the DH domain, and a hydrophobic packing in the middle (Figure 1D). At the C-terminal end, the DH domain Asp^2193^ forms hydrogen bonds with the backbone nitrogen and the side chain of RhoA Arg^68^. RhoA Leu^69^ packs in between DH domain residues Cys^2105^, Val^2196^, Ile^2189^ and Val^2192^, whereas the aromatic ring of Tyr^66^ packs against Ile^2189^ and the methyl group of Thr^2155^. At the N-terminal end of switch I, Gln^2152^ binds the backbone carbonyl of RhoA Asp^59^.

### No contacts between the AKAP-Lbc PH domain and RhoA

For some RhoGEFs such as LARG (29% sequence identity with AKAP-Lbc over the DH–PH domain) the PH domain contributes to GEF activity. The AKAP-Lbc PH domain made no contacts with RhoA in the crystal structure (Figures 1B, 2A and 2B). Comparison with the structure of RhoA–LARG (PDB code 1X86) [23] reveals that residues crucial for the involvement of the PH domain in stabilizing the RhoA–RhoGEF complex are not conserved in AKAP-Lbc (Figures 2C, 2D and 2E). LARG, p115 and PRG all have a TXS(D/E) motif at the start of the PH domain helix (residues 1116–1119 in LARG) which interacts with RhoA Glu^97^. In AKAP-Lbc this is replaced by an SXEE sequence. The replacement of Ser^2323^ with glutamic acid in AKAP-Lbc is presumably unfavourable for binding the also negatively charged RhoA Glu^97^. Indeed, a LARG S1118D mutation cancelled the additional contribution of the LARG PH domain to nucleotide exchange, as did an E1023A mutation [23].

One proposal for the mechanism by which a RhoGEF PH domain can contribute to GEF activity is stabilization of the long C-terminal helix of the DH domain (αI). This helix forms crucial binding interactions with the GTPase switch II loop. Crucial residues for this stabilization are not conserved in AKAP-Lbc. In LARG, RhoA Arg^68^ is bound by Glu^982^ and Asn^983^ (Figures 2C and 2D). Asn^983^ is replaced with Lys^2001^ in AKAP-Lbc which cannot form the same binding interaction and RhoA Arg^68^ is instead bound to Asp^2193^, much closer to the N-terminus of αI (Figure 2B). Furthermore, the binding of Arg^68^ to the LARG DH domain is made possible by the curvature and stabilization of the αI C-terminus induced by close association to the PH domain (Figures 2D and 2E). This association is also required for proximity of the PH domain helix to RhoA Glu^97^. Although the crucial LARG residue Arg^986^ is conserved in AKAP-Lbc (Arg^2204^), the other residues are entirely non-conserved. For example, the LARG PH domain residues Tyr^1039^ and Glu^1023^ are replaced with Gln^2255^ and the shorter Asp^2239^ in AKAP-Lbc. The result is that the binding arrangement is quite different in AKAP-Lbc and results in an entirely straight long α-helix (αI) with four turns of this α-helix separating the residues interacting with switch II from the residues interacting with the PH domain (Figures 2A and 2B).

### AKAP-Lbc PH domain is not required for catalysis of RhoA nucleotide exchange

**Figure 3:**
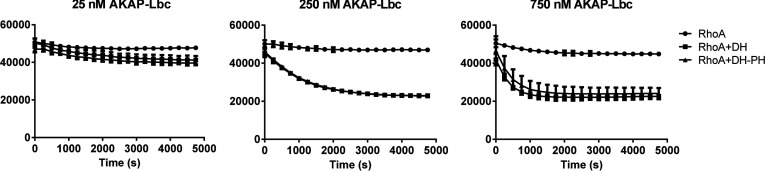
The PH domain does not influence the DH-domain-induced GEF on RhoA The rate of exchange of bound MANT-GDP for unlabelled GMP-PNP in the absence and presence of either the DH domain or the combined DH–PH domain of AKAP-Lbc. The decrease in fluorescence of MANT-GDP upon leaving RhoA was measured over time. The *y*-axis shows arbitrary fluorescence units. Measurements are plotted as the means±S.D. from three replicates.

To investigate whether for our constructs the AKAP-Lbc PH domain contributes to catalysis of nucleotide exchange on RhoA we cloned an expression construct containing just the DH domain (residues 1976–2211, terminating at the end of αI) for comparison with the combined DH–PH domain (Table 3). We performed nucleotide-exchange assays using the decrease in fluorescence when MANT-GDP moves into a solvent environment after being bound to a protein [47]. The rate of exchange of RhoA–MANT-GDP for RhoA–GTP was measured in the presence of 25 nM, 250 nM and 750 nM of either the AKAP-Lbc DH–PH domain or the DH domain alone (Figure 3). The rate increased with increasing concentrations of DH–PH or DH domain alone, but at all three concentrations measured the rate was unaffected by the presence of the PH domain.

**Figure 4:**
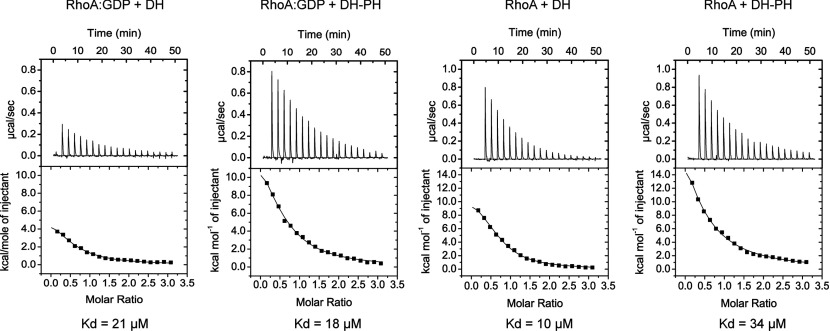
ITC measurements of the dissociation constants (*K*_d_) between RhoA and the AKAP-Lbc DH–PH domain or the AKAP-Lbc DH domain, in the presence or absence of GDP

To analyse further any possible role for the PH domain in the interactions with RhoA we compared the dissociation constants for the complexes of RhoA with the DH–PH domain or with the DH domain alone. We also wanted to measure dissociation constants with RhoA–GDP and with apo-RhoA to see whether AKAP-Lbc would have greater affinity for apo-RhoA as has been observed previously for other RhoGEFs [48]. Binding affinities were measured by ITC. For measurements in the presence of GDP, all proteins were dialysed beforehand against buffer containing GDP, and the measurements were performed in this buffer to counteract any effects of nucleotide binding. For measurements in the absence of GDP, any bound nucleotide was removed from RhoA beforehand by phosphatase treatment. The measurements revealed similar dissociation constants (*K*_d_) within experimental error of ~20 μM for RhoA against either the DH–PH domain or the DH domain alone, in the presence or absence of GDP (Figure 4).

### Changes in the conformation of the DH domain upon binding to RhoA

The N-terminus of some DH domains contains a ‘GEF switch’ region [43], a short sequence motif just N-terminal to the first α-helix (αA) of the DH domain. For both LARG and p115-RhoGEF, this region was shown to be important for catalysis of nucleotide exchange [23,43]. Deletion of the GEF switch from LARG or mutation of the conserved tryptophan residue at the centre of the motif (LARG Trp^769^) to alanine or aspartate reduced GEF activity to 15–20% of wild-type [23]. Furthermore, LARG E790G also reduced GEF activity to 15–20%, suggesting that the critical factor is its interaction with RhoA Tyr^34^.

**Figure 5:**
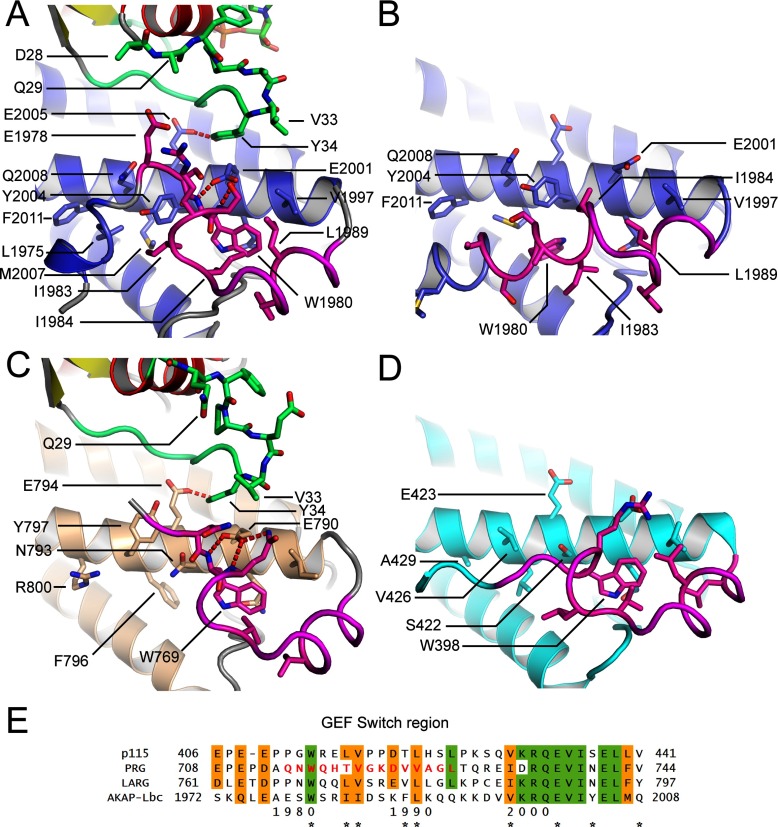
Comparison of the GEF switch region at the N-terminus of the AKAP-Lbc DH domain with the equivalent regions in LARG and p115-RhoGEF (**A**) The conformation of the AKAP-Lbc DH domain GEF switch region (coloured in magenta) when bound to RhoA. RhoA is coloured with red α-helices and yellow β-strands with its switch I region in green. The DH domain of AKAP-Lbc is coloured blue. (**B**) The conformation of the DH domain when not bound to RhoA. (**C** and **D**) The conformation of the equivalent regions in LARG when bound to RhoA (**C**) and p115-RhoGEF when not bound to RhoA (**D**). (**E**) Sequence alignment of the GEF switch regions of AKAP-Lbc and three homologous RhoGEF domains. Residues important for the packing of the GEF switch region are marked with asterisks below the alignment.

The GEF switch region of AKAP-Lbc's DH domain (Figure 5A) adopts a similar conformation to that of LARG (Figure 5C). RhoA Tyr^34^ packs against the conserved Glu^2001^ (LARG Glu^790^), whereas forming a hydrogen bond to the also conserved Glu^2005^ (LARG Glu^794^). The tryptophan appears to orient the backbone of the GEF switch region so that Glu^2001^ can form hydrogen bonds to two backbone nitrogens (Figures 5A and 5C). This presumably stabilizes the conformation of Glu^2001^ in a conformation favourable for interaction with RhoA Tyr^34^.

To analyse conformational changes in the DH domain on binding we crystallized the DH domain alone in the absence of RhoA (Table 1), revealing a significant movement of the GEF switch region (Figure 5B), which adopts an α-helical conformation with Trp^1980^ itself binding in a different pocket on the first DH domain α-helix. This we refer to as the inactive conformation, in which the position occupied by Trp^1980^ in the active conformation is now occupied by Ile^1984^ and Ile^1983^ (Figure 5B). We cannot rule out that the inactive α-helical conformation is induced by the N-terminal protein tag which is at the start of this α-helix. However, we note that the region around Trp^1980^ is weakly predicted to be α-helical by the PsiPred server [49], suggesting that a conformational exchange in and out of an α-helix induced by the absence or presence of RhoA is a realistic model. In this inactive conformation, the critical Glu^2001^ can no longer bind to RhoA (Figure 4B). In contrast with AKAP-Lbc, in the structure of p115-RhoGEF in the absence of RhoA [43], the GEF switch maintains an active conformation (Figure 5D).

### Potential for targeting small molecules against the GEF activity of AKAP-Lbc

**Figure 6:**
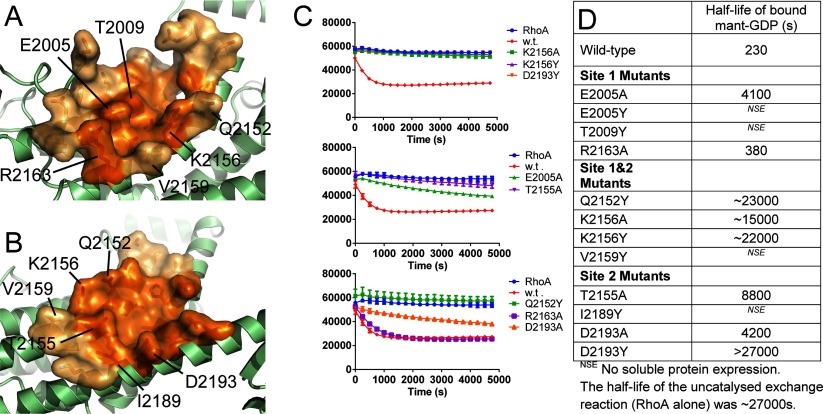
Surface pockets on the AKAP-Lbc DH domain are potentially suitable for binding of protein–protein interface inhibitors (**A**) Site 1 which binds the switch I loop of RhoA. (**B**) Site 2 which binds the switch II loop of RhoA. The core of each pocket containing the residues in direct contact with RhoA is coloured dark orange, whereas the surrounding region of the pocket is coloured light orange. Sites of mutations made are indicated (**C**). Nucleotide-exchange assays for DH domain mutants compared with wild-type. (**D**) Approximate half-lives of RhoA–MANT-GDP based on fitting the curves in (**C**) to an exponential decay.

There are two shallow pockets (Figures 6A and 6B) on the surface of the DH domain which are involved in the binding of the RhoA switch I and switch II loops (Site 1 and Site 2). An inhibitor bound at either or both of these sites might prevent binding and activation of RhoA. To validate the functional consequences of small molecules binding at these sites we created 12 point mutations, either to alanine (smaller) or to tyrosine (larger) on the surface of the DH domain around the pockets (Figure 6D). Four of these mutant proteins (all replaced with a tyrosine residue) failed to generate soluble protein expression. The remaining eight were purified and analysed in the GEF assay (Figure 6C). The curves were fitted to an exponential equation to calculate a half-life for bound MANT-GDP at 750 nM AKAP-Lbc. Mutation R2163A (site 1) showed activity equivalent to wild-type. The remaining mutations to alanine all showed decreased exchange activity consistent with reduced affinity of binding, whereas the mutations to tyrosine all essentially abolished the GEF activity of AKAP-Lbc. This suggests that binding of small molecules at these sites would also abolish GEF activity.

## DISCUSSION

The RhoGEF activity of AKAP-Lbc is potentially interesting for therapeutic intervention. With only modest binding affinity for RhoA, development of a RhoA-competitive inhibitor should be possible. Furthermore, since the PH domain is not involved in nucleotide exchange, any compound screening programme need only be concerned with the DH domain. With the low sequence conservation between AKAP-Lbc and related RhoGEFs, it is likely that AKAP-Lbc-specific inhibitors could be generated.

We analysed the two potential binding pockets for small molecules using SiteMap (Schrödinger) which takes into account the volume of the pocket, degree of enclosure and degree of hydrophobicity. AKAP-Lbc had SiteMap Dscores of 0.74 and 0.83 respectively for the switch I and switch II binding pockets, with pocket volumes of 133 and 145 Å^3^. These were the top two sites (by Dscore) that were identified. By comparison with a well-known druggable protein with a larger binding side, using the same methodology the protein kinase Aurora A (PDB code 1MQ4) has a Dscore of 0.96 with a pocket volume of 591 Å^3^. Bromodomains for which small molecule ligands have been identified span a range of Dscores from >1.0 to 0.62 [50]. Within this range, two exemplars BRD4(1) and BAZ2B had median Dscores of 0.93 and 0.62 respectively [50]. For comparison, using the same SiteMap settings as for AKAP-Lbc, we calculated Dscores of 0.90 and 0.66 for BRD4(1) (PDB code 3MXF) and BAZ2B (PDB code 3G0L), with pocket volumes of 125 and 109 Å^3^. In conclusion, AKAP-Lbc appears at least as druggable as bromodomains for which many ligands have already been identified [51].

Finally, the analysis of the sequence and structural reasons for the lack of involvement of the AKAP-Lbc PH domain in GEF activity may allow useful predictions to be made for other RhoGEFs, of which there are approximately 72 human members having the DH–PH domain architecture [15].

## Online data

Supplementary data
