# Supplementary material for: The crystal structure of the RhoA–AKAP-Lbc DH–PH domain complex
Source: Biochem J. 2014 Nov 14;464(Pt 2):231–9. doi: 10.1042/BJ20140606 (PMC4232260; doi:10.1042/BJ20140606)
Supplement: Supplementary data [file bj4640231ntsadd.pdf]

The Crystal Structure of the RhoA : AKAP-Lbc DH-PH Domain Complex

Supplementary Material

Contents

Protein sequences of the constructs used 2
RhoA 2
AKAP-Lbc DH-PH domain 2
AKAP-Lbc DH domain 2
Figure S1 - Sequence alignment of selected RhoGEF domains 3

Protein sequences of the constructs used

RhoA

SMAAIRKKLVIVGDGACGKTCLLIVFSKDQFPEVYVPTVFENYVADIEVDGKQVELALWDTAGQEDYDRLRPLSYPDTD
VILMCFSIDSPDSLENIPEKWTPEVKHFCPNVPIILVGNKKDLRNDEHTRRELAKMKQEPVKPEEGRDMANRIGAFGYM
ECSAKTKDGVREVFEMATRAALQARRG

AKAP-Lbc DH-PH domain

SMSKQLEAESWSRIIDSKFLKQQKKDVVKRQEVIYELMQTEFHHVRTLKIMSGVYSQGMMADLLFEQQMVEKLFPCL
DELISIHSQFFQRILERKKESLVDKSEKNFLIKRIGDVLVNQFSGENAERLKKTYGKFCGQHNQSVNYFKDLYAKDKRFQA
FVKKKMSSSVVRRLGIPECILLVTQRITKYPVLFQRILQCTKDNEVEQEDLAQSLSLVKDVIGAVDSKVASYEKKVRLNEIYT
KTDSKSIMRMKSGQMFAKEDLKRKKLVRDGSVFLKNAAGRLKEVQAVLLTDILVFLQEKDQKYIFASLDQKSTVISLKKLI
VREVAHEEKGLFLISMGMTDPEMVEVHASSKEERNSWIQIIQDTINTLNRD

AKAP-Lbc DH domain

SMEAESWSRIIDSKFLKQQKKDVVKRQEVIYELMQTEFHHVRTLKIMSGVYSQGMMADLLFEQQMVEKLFPCLDELISI
HSQFFQRILERKKESLVDKSEKNFLIKRIGDVLVNQFSGENAERLKKTYGKFCGQHNQSVNYFKDLYAKDKRFQAFVKKK
MSSSVVRRLGIPECILLVTQRITKYPVLFQRILQCTKDNEVEQEDLAQSLSLVKDVIGAVDSKVASYEKKVRLNEIYTK

Figure S1 - Sequence alignment of selected RhoGEF domains

```
p115-RhoGEF     ---------------------------------------DEGEPGRSGLELEPE-EPPGW 413
PDZ-RhoGEF      ---------------------------------------LLEDDLGQLSDLEPEPDAQNW 716
LARG            ---------------------------------------GESQSEDEQFENDLETDPPNW 769
AKAP-Lbc        ---------------------------------------------------SKQLEAESW 1980
                                                                   . : :. .*

p115-RhoGEF     RELVPPDTLHSLPKSQVKRQEVISELLVTEAAHVRMLRVLHDLFFQPMAECLFFPLEELQ 473
PDZ-RhoGEF      QHTVGKDVVAGLTQREIDRQEVINELFVTEASHLRTLRVLDLIFYQRMKKENLMPREELA 776
LARG            QQLVSREVLLGLKPCEIKRQEVINELFYTERAHVRTLKVLDQVFYQRVSREGILSPSELR 829
AKAP-Lbc        SRIIDSKFLKQQKKDVVKRQEVIYELMQTEFHHVRTLKIMSGVYSQGMMADLLFEQQMVE 2040
                 . :  . :       :.***** **: **  *:* *:::  :: * :    ::  . :

p115-RhoGEF     NIFPSLDELIEVHSLFLDRLMKRRQES-------GYLIEEIGDVLLARFDGAEGSWFQKI 526
PDZ-RhoGEF      RLFPNLPELIEIHNSWCEAMKKLREE--------GPIIKEISDLMLARFDGPAREELQQV 828
LARG            KIFSNLEDILQLHIGLNEQMKAVRKRNE------TSVIDQIGEDLLTWFSGPGEEKLKHA 883
AKAP-Lbc        KLFPCLDELISIHSQFFQRILERKKESLVDKSEKNFLIKRIGDVLVNQFSGENAERLKKT 2100
                .:*. * :::.:*    : :   ::.          :*..*.: ::  *.*   . :::

p115-RhoGEF     SSRFCSRQSFALEQLKAKQRKDPRFCAFVQEAESRPRCRRLQLKDMIPTEMQRLTKYPLL 586
PDZ-RhoGEF      AAQFCSYQSIALELIKTKQRKESRFQLFMQEAESHPQCRRLQLRDLIISEMQRLTKYPLL 888
LARG            AATFCSNQPFALEMIKSRQKKDSRFQTFVQDAESNPLCRRLQLKDIIPTQMQRLTKYPLL 943
AKAP-Lbc        YGKFCGQHNQSVNYFKDLYAKDKRFQAFVKKKMSSSVVRRLGIPECILLVTQRITKYPVL 2160
                 . **. :  ::: :*    *: **  *::.  * .  *** : : *    **:****:*

p115-RhoGEF     LQSIGQNTEEPT-EREKVELAAECCREILHHVNQAVRDMEDLLRLKDYQRRLDLSHLRQS 645
PDZ-RhoGEF      LESIIKHTEGGTSEHEKLCRARDQCREILKYVNEAVKQTENRHRLEGYQKRLDATALERA 948
LARG            LDNIAKYTEWPT-EREKVKKAADHCRQILNYVNQAVKEAENKQRLEDYQRRLDTSSLKLS 1002
AKAP-Lbc        FQRILQCTKDNEVEQEDLAQSLSLVKDVIGAVDSKVASYEKKVRLNEIYTKTDSKSIMRM 2220
                :: * : *:    *:*.:  : .  ::::  *:. * . *.  **:    : * . :

p115-RhoGEF     SDPMLSEFKNLDITKKKLVHEGPLTWRVTKDKAVEVHVLLLDDLLLLLQRQDERLLLKSH 705
PDZ-RhoGEF      SNPLAAEFKSLDLTTRKMIHEGPLTWRISKDKTLDLHVLLLEDLLVLLQKQDEKLLLKCH 1008
LARG            EYPNVEELRNLDLTKRKMIHEGPLVWKVNRDKTIDLYTLLLEDILVLLQKQDDRLVLRCH 1062
AKAP-Lbc        KS--GQMFAKEDLKRKKLVRDGSVFLKNAAGRLKEVQAVLLTDILVFLQEKDQKYIFASL 2278
                .      : . *:. :*::::*.:  :   .:  :: .:** *:*::**.:*:: :: .

p115-RhoGEF     SRTLTPTPDGKTMLRPVLRLTSAMTREVATDHKAFYVLFTWDQ-EAQIYELVAQTVSERK 764
PDZ-RhoGEF      SKTAVGSSDSKQTFSPVLKLNAVLIRSVATDKRAFFIICTSKLGPPQIYELVALTSSDKN 1068
LARG            SKILASTADSKHTFSPVIKLSTVLVRQVATDNKALFVISMSDN-GAQIYELVAQTVSEKT 1121
AKAP-Lbc        DQKST-----------VISLKKLIVREVAHEEKGLFLISMGMT-DPEMVEVHASSKEERN 2326
                .:  .           *: *.  : *.** :.:.:::        .:: *: * : .::.

p115-RhoGEF     NWCALITETAGSLKVPAPASRPKPRPSPSSTREPLLSSSE------------NGNGGRET 812
PDZ-RhoGEF      TWMELLEEAVRNATRHPGAAPMPVHPPPPGPREPAQQGPTPSRVELDDSDVFHGEPEPEE 1128
LARG            VWQDLICRMAASVKEQSTKPIPLPQSTPGEGDNDEEDPSKLKEEQHG--ISVTGLQSPDR 1179
AKAP-Lbc        SWIQIIQDTINTLNRD
                 *  ::     . .
```

Protein	Official Symbol	Genbank ID
p115-RhoGEF	ARHGEF1	NP_945353.1
PDZ-RhoGEF	ARHGEF11	NP_055599.1
LARG	ARHGEF12	NP_056128.1
AKAP-Lbc	AKAP13	NP_006729.4
